# Twitter Analysis of the Nonmedical Use and Side Effects of Methylphenidate: Machine Learning Study

**DOI:** 10.2196/16466

**Published:** 2020-02-24

**Authors:** Myeong Gyu Kim, Jungu Kim, Su Cheol Kim, Jaegwon Jeong

**Affiliations:** 1 Graduate School of Clinical Pharmacy CHA University Pocheon Republic of Korea; 2 Department of Psychiatry Anam Hospital Seoul Republic of Korea

**Keywords:** methylphenidate, social media, Twitter, prescription drug misuse, drug-related side effects and adverse reactions, machine learning, support vector machine

## Abstract

**Background:**

Methylphenidate, a stimulant used to treat attention deficit hyperactivity disorder, has the potential to be used nonmedically, such as for studying and recreation. In an era when many people actively use social networking services, experience with the nonmedical use or side effects of methylphenidate might be shared on Twitter.

**Objective:**

The purpose of this study was to analyze tweets about the nonmedical use and side effects of methylphenidate using a machine learning approach.

**Methods:**

A total of 34,293 tweets mentioning methylphenidate from August 2018 to July 2019 were collected using searches for “methylphenidate” and its brand names. Tweets in a randomly selected training dataset (6860/34,293, 20.00%) were annotated as positive or negative for two dependent variables: nonmedical use and side effects. Features such as personal noun, nonmedical use terms, medical use terms, side effect terms, sentiment scores, and the presence of a URL were generated for supervised learning. Using the labeled training dataset and features, support vector machine (SVM) classifiers were built and the performance was evaluated using F_1_ scores. The classifiers were applied to the test dataset to determine the number of tweets about nonmedical use and side effects.

**Results:**

Of the 6860 tweets in the training dataset, 5.19% (356/6860) and 5.52% (379/6860) were about nonmedical use and side effects, respectively. Performance of SVM classifiers for nonmedical use and side effects, expressed as F_1_ scores, were 0.547 (precision: 0.926, recall: 0.388, and accuracy: 0.967) and 0.733 (precision: 0.920, recall: 0.609, and accuracy: 0.976), respectively. In the test dataset, the SVM classifiers identified 361 tweets (1.32%) about nonmedical use and 519 tweets (1.89%) about side effects. The proportion of tweets about nonmedical use was highest in May 2019 (46/2624, 1.75%) and December 2018 (36/2041, 1.76%).

**Conclusions:**

The SVM classifiers that were built in this study were highly precise and accurate and will help to automatically identify the nonmedical use and side effects of methylphenidate using Twitter.

## Introduction

Methylphenidate is a stimulant that is widely used for treating attention deficit hyperactivity disorder (ADHD) [1]. It was approved for use in children and adolescents, and recently for adult ADHD, in several countries including the United States [2]. The use of methylphenidate is increasing worldwide [3]. Although methylphenidate is considered safe to use when taken as prescribed, it does have the potential for abuse because of its focus-enhancing, appetite-reducing, and euphoric effects [4,5]. In particular, more and more students are taking methylphenidate for academic purposes, calling it a “smart drug” or “study drug” [6-10]. A previous systematic review of 21 studies showed that 5%-9% of grade school- and high school-age children, as well as 5%-35% of college-age students, misused stimulants such as methylphenidate and amphetamines [11]. In addition, children and adolescents take methylphenidate to stay up for parties and experience euphoria [8,9]. Due to the potential abuse of methylphenidates, many countries classify and control the drug legally [3,4].

Children and adult ADHD patients can benefit from the therapeutic effects of methylphenidate with few side effects when the drug is used as prescribed [1]. However, studies have shown that children and adolescents who use methylphenidate to treat ADHD have a 60% higher risk of sleep disorders and a 266% higher risk of loss of appetite than those in control groups [12]. In addition, children and adolescents with ADHD often have comorbid mood disorders and anxiety disorders, and the use of stimulants such as methylphenidate could exacerbate these comorbidities [13]. Sometimes methylphenidate can cause hallucinations and delusions [14,15]. The rates of adverse drug reactions to methylphenidate, including agitation, irritability, and elevated heart rate, increase when it is abused [1,4,10]. A total of 40% of 394 toxic exposures of methylphenidate reported in Denmark involved recreational use [3]. Central nervous system and constitutional symptoms, such as anorexia, fatigue, and insomnia, were reported in 263 out of 323 cases (81.4%), and cardiovascular symptoms, such as arrhythmias, hypertension, and myocardial infarctions, were reported in 227 out of 323 (70.3%) of the symptomatic cases [3]. The number of emergency room visits due to nonmedical use of ADHD medications nearly doubled between 2005 and 2010, from 5085 to 9181 [16]. Thus, the abuse of methylphenidate has become a public health problem.

It is important to know the state of abuse and side effects of stimulants. Most studies of methylphenidate abuse have been conducted using surveys [11]. The survey method is appropriate for investigating the status and motivation of nonmedical use; however, it is limited by efforts to conceal abuse, and some potential subjects may decline to participate in the study because of the fear of being discovered [9]. Postmarketing surveillance studies using spontaneous reporting systems, such as the US Food and Drug Administration Adverse Event Reporting System, are suitable for analyzing the incidence and types of side effects caused by the use of methylphenidates but are limited in their ability to assess the current state of methylphenidate use.

As a new means of investigation, social networking services (SNSs) have begun to get attention in overcoming such challenges. Nowadays, it has become common to share one's thoughts, search for opinions, and interact with people with similar ideas through SNSs. In contrast to other SNSs, Twitter delivers most of its content as text rather than images, and many tools have been developed to analyze the content or emotions implicit in tweets. Consequently, it is relatively easy to analyze Twitter users' experiences or thoughts on a particular topic. Health researchers are increasingly using Twitter to analyze content about various topics (56%), as well as for surveillance (26%), engagement (14%), subject recruitment (7%), intervention (7%), and network analysis (4%) [17]. From 2010 to 2015, the number of health researchers using Twitter increased almost 20-fold [17]. Some studies have been conducted to analyze tweets about sentiment toward marijuana or tobacco smoking [18,19]. Recently, studies have been conducted that used machine learning to analyze Twitter’s big data repository. A study developed a classification program that could automatically detect opioid users from Twitter [20]. Twitter and other SNSs are mainly utilized by younger users, and these groups are more likely to be diagnosed with ADHD or to abuse methylphenidate [21]. The aim of this study was to analyze tweets about nonmedical use and side effects of methylphenidate using a machine learning approach.

## Methods

### Study Design

**Figure 1:**
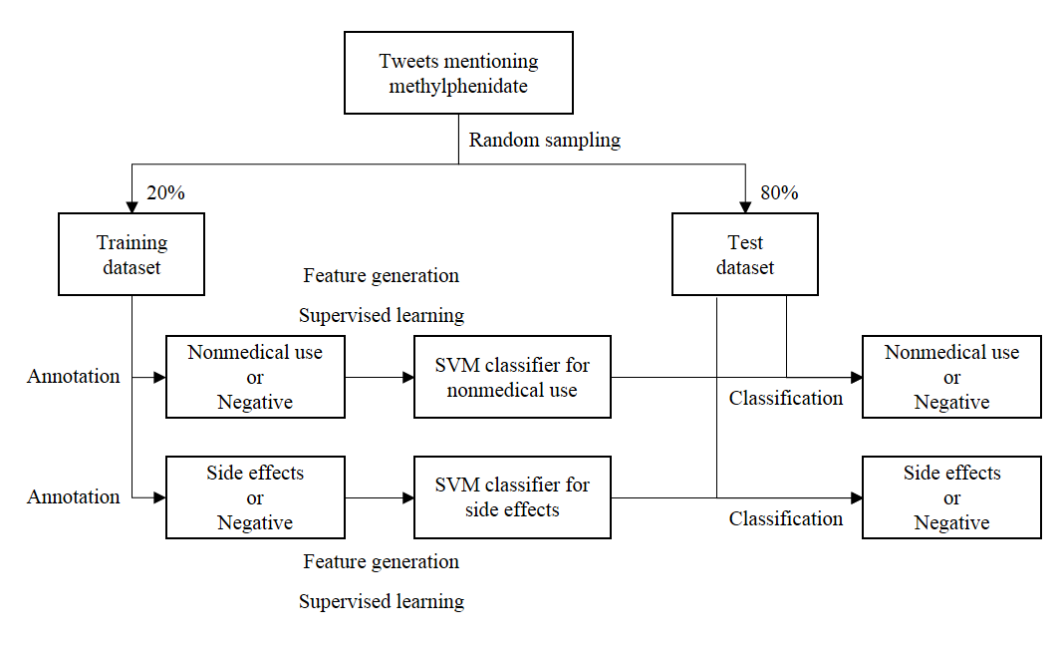
Schematic diagram of the study design. SVM: support vector machine.

The steps in this study were conducted in the following order: tweet collection, manual annotation, feature generation, supervised learning, and classification of the test dataset (see Figure 1). The study was exempted from Institutional Review Board review (201909-HR-067-01).

### Tweet Collection

Tweets mentioning methylphenidate from August 2018 to July 2019 were collected using the Twitter premium search application programming interface and Python, version 3.7.4 (Python Software Foundation). The following search terms for methylphenidate and its brand names were used: “methylphenidate,” “Aptensio,” “Biphentin,” “Concerta,” “Daytrana,” “Equasym,” “Jornay,” “Medikinet,” “Metadate,” “Methylin,” “Quillichew,” “Quillivant,” “Ritalin,” and “Rubifen.” This study did not cover the drug Adhansia because it was only approved in February 2019. Retweets were also collected if the user added their own text to an original tweet that contained the search terms. Duplicate tweets were removed, and only tweets written in English were used.

### Annotation

Among the collected tweets (N=34,293), 6860 (20.00%) were randomly selected as a training dataset. First, two annotators manually identified tweets mentioning first-hand experience. Tweets about drugs other than methylphenidate, song lyrics, humor, news, study results, or someone else’s experience were annotated as *non-first-hand experience* [22]. Second, tweets about first-hand experience were classified as *nonmedical use*, *side effects*, and *other*. Tweets could be classified as pertaining to both nonmedical use and side effects. Finally, tweets were labeled positive and negative for two dependent variables: nonmedical use and side effects. Due to the nature of the short length of the text, it was often difficult to determine whether the drug was used nonmedically. In such cases, tweet threads or past tweets were checked to determine whether the user had ever been diagnosed with a condition that required methylphenidate. Interannotator agreement was assessed by Cohen kappa values [23,24], and any disagreements were resolved by discussion among psychiatrists.

### Feature Generation

Several features for supervised learning were generated: personal noun, nonmedical use terms, medical use terms, side effect terms, sentiment scores, and the presence of a URL (see Table 1).

**Table 1:** Features for supervised learning.

Feature and subfeature		Included terms
**Personal noun**		
	First person	i, i’, my, me, mine, myself, im, iam
	Second person	you, you’, your, yours, yourself, ur
	Third person	he, he’, his, him, himself, she, she’, her, hers, herself, they, they’, their, theirs, them, themselves
	Others	boy, boyfriend, child, children, daughter, friend, girl, girlfriend, husband, kid, son, wife
**Nonmedical use terms**		
	General terms	abus, misus
	Alternative motives	allnight, assign, clean, colleg, cram, diet, essay, exam, examin, final, focus, highschool, homework, loss, midnight, midterm, nighter, overnight, paper, paperwork, parti, project, quiz, recreat, school, shift, studi, studyin, test, work, write
	Overdose	double, extra, overdos, overus, pop
	Alternative route of administration	crush, inhal, inject, rail, sniff, sniffin, snort, snortin
	Seeking	need, want, wish
	Obtaining	buy, sell, share, steal, trade
	Coingestion	alcohol, beer, bird, booz, bull, caffeine, cocain, coffe, coke, crack, crystal, energi, energydrink, espresso, heroin, lsd, marijuana, monster, pot, redbul, seed, shot, tequila, vodka, weed, wine, xtc
Medical use terms		addadhd, adhd, defici, diagnos, diagnosis, disord, narcolepsy, narcolept, prescribe, prescript
**Side effect terms**		
	General terms	side, sideeffect, advers
	Loss of appetite	anorexia, appetit, ate, eat, eaten, eatin, food, hungry, lbs, meal, skinni, slim, starv, thin, underweight, weight
	Sleep problems	asleep, awak, insomnia, insomniac, sleep, sleepi, sleepless, slept, tire
	Psychiatric problems	anxieti, anxious, depress, jitter, jitteri, nervous, obsess, panic, restless, shaki, shakin, tens, tension, worri, zombie
	Heart problems	beat, heart, heartbeat, heartrat, palpit
	Gastrointestinal problems	diarrhea, dri, nausea, nauseat, nauseous, stomach, throw, thrown, vomit
	Neurological problems	dizzi, head, headach, lighthead, migrain
	Sweating	hot, sweat, sweatin
	Eye problems	blurri, vision, visual
Sentiment scores		N/A^a^
Presence of a URL		N/A

^a^Not applicable.

### Personal Noun

The *personal noun* feature was generated to identify tweets mentioning first-hand experience. Personal nouns were grouped into one of four categories: first person, second person, third person, and others. *Others* included terms that could be used to describe someone else’s experience. Due to frequent nonstandard grammatical usage on Twitter, common modifications such as “im,” “iam,” and “ur” were also included.

### Nonmedical Use Terms and Medical Use Terms

Prior to generating the features of nonmedical use terms, natural language processing was performed using the *tm* package from R, version 3.6.1 (The R Foundation), and RStudio. First, numbers, punctuation, and stop words, such as “the,” “is,” “and,” etc, were removed from the text of the tweets. Later, the tweet text was divided by word unit. Words were converted to their stems using the *stemDocument* function from R, and the frequency of word appearance in each tweet was described in a term-document matrix.

The counts of nonmedical use terms in individual tweets was used as a feature and this feature included seven subfeatures: general terms, alternative motives, overdose, alternative route of administration, seeking, obtaining, and coingestion. Terms were selected based on similar studies [25,26]. Further, words related to nonmedical use were added by comparing words that appeared at a frequency of 5% or higher in tweets annotated as *nonmedical use* or *negative*. The counts of medical use terms were used to exclude medical use of methylphenidate for the treatment of ADHD or narcolepsy.

### Side Effect Terms

The counts of side effect terms were generated as a feature after natural language processing as described above. Side effect terms included general terms, loss of appetite, sleep problems, psychiatric problems, heart problems, gastrointestinal problems, neurological problems, sweating, and eye problems. As in the selection of nonmedical terms, the terms included were added by reference to previous studies [25] or by comparing the words that appeared in the training dataset.

### Sentiment Scores

Sentiment scores were used as a feature because users often write polarized sentimental words when mentioning drug abuse or side effects. Sentiment scores were calculated by adding the number of positive words (each counting as +1) and the number of negative words (each counting as -1) appearing in a tweet. The Liu and Hu opinion lexicon dictionary, which contains 6800 positive and negative words in the English language, was used for sentiment analysis [27]. Some negative words, such as “wtf,” were added to the dictionary.

### Presence of a URL

This feature was created to identify retweets containing the user’s content or link to another website. Links to other websites were usually news or study results.

### Supervised Learning

A support vector machine (SVM) with a radial basis function kernel was trained to classify nonmedical use and side effects using the *e1071* package from R and RStudio. Two parameters of the SVM—cost and gamma—were tuned to achieve a better performance. Because the training dataset had a very large number of negative samples, 10-fold cross-validation was performed on the training data, and inverse weights were assigned to positive and negative samples to compensate for the imbalance [28]. Due to the imbalance in the data, the F_1_ score (ie, harmonic mean of precision and recall) was used instead of accuracy to measure the performance of the SVM classifier. Precision, recall, accuracy, and F_1_ score were calculated as follows:

Precision = TP / (TP + FP) (1)

Recall = TP / (TP + FN) (2)

Accuracy = (TP + TN) / (TP + TN + FP + FN) (3)

F_1_ = (2 × Precision × Recall) / (Precision + Recall) (4)

True positives (TP), false positives (FP), true negatives (TN), and false negatives (FN) were calculated by comparing annotated results and predicted results.

### Classification of Test Dataset

The SVM classifier separated the test dataset into *nonmedical use* or *side effects* and *negative*. Tweets about nonmedical use and side effects were counted. The number of tweets about nonmedical use of methylphenidate each month was determined to examine the correlation with the school term.

## Results

From August 2018 to July 2019, 36,578 tweets were collected using predetermined search terms. Tweets containing the words “Ritalin” and “Concerta” were the most frequent (27,635/36,578, 75.55%, and 5485/36,578, 15.00%, respectively). In total, 34,293 nonduplicated tweets were ultimately selected: 6860 (20.00%) in the training dataset and 27,433 (79.99%) in the test dataset.

Among the 6860 tweets in the training dataset, 2108 (30.73%) mentioned first-hand experience, including 356 about nonmedical use (5.19%) and 379 about side effects (5.52%). A total of 20 tweets (0.29%) were annotated as pertaining to both nonmedical use and side effects. Cohen kappa values were .73 and .75 for nonmedical use and side effects, respectively, which means there was substantial agreement between the two annotators.

The classification performance of SVM classifiers, expressed as an F_1_ score, was 0.547 for nonmedical use and 0.733 for side effects (see Tables 2 and 3). The low recall of the SVM classifier for nonmedical use, despite its high precision, was responsible for its low F_1_ score. Each feature contributed to improvement of SVM classifiers (see Tables 2 and 3). With the exception of nonmedical use and side effect terms, F_1_ scores were the lowest when the feature *personal noun* was excluded.

**Table 2:** Classification performance of support vector machine (SVM) classifiers for nonmedical use of methylphenidate.

SVM classifier	F_1_ score for *nonmedical use*	F_1_ score for *negative*	Precision	Recall	Accuracy
Final model	0.547	0.983	0.926	0.388	0.967
Without nonmedical use terms	0.145	0.975	0.933	0.079	0.952
Without medical use terms	0.506	0.982	0.925	0.348	0.965
Without personal noun	0.233	0.976	0.857	0.135	0.954
Without sentiment scores	0.420	0.979	0.833	0.281	0.960
Without a URL	0.526	0.982	0.923	0.368	0.966

**Table 3:** Classification performance of support vector machine (SVM) classifier for side effects of methylphenidate.

SVM classifier	F_1_ score for *side effects*	F_1_ score for *negative*	Precision	Recall	Accuracy
Final model	0.733	0.987	0.920	0.609	0.976
Without side effect terms	0.316	0.976	0.880	0.193	0.954
Without personal noun	0.388	0.978	0.887	0.248	0.957
Without sentiment scores	0.571	0.982	0.918	0.414	0.966
Without a URL	0.722	0.987	0.922	0.594	0.975

From the test dataset (n=27,433), 361 tweets (1.32%) about the nonmedical use and 519 tweets (1.89%) about the side effects of methylphenidate were identified using SVM classifiers. A total of 21 tweets (0.08%) were classified as pertaining to both nonmedical use and side effects. Examples of tweets, paraphrased to ensure anonymity, classified as nonmedical use included “When I was young I snorted my Concerta only for the head rush” and “Time to pop the Ritalin I been keeping.” Paraphrased tweets classified as side effects included “Worst 9 days of my life. I thought Ritalin would calm the anxiety part of the ADHD. But it makes me a short-fused angry psycho who burst into tears spontaneously” and “Seizures, hallucinations, paranoia is all Ritalin brought me. The side effects still bother me.”

**Figure 2:**
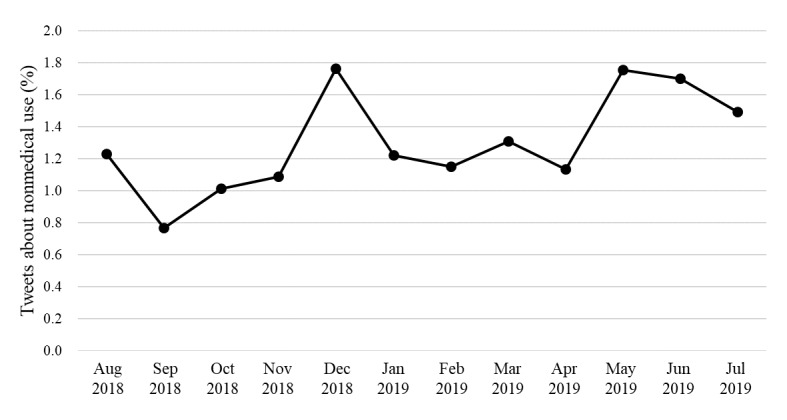
Distribution of tweets about nonmedical use of methylphenidate, by month.

The monthly proportion of tweets about the nonmedical use of methylphenidate is shown in Figure 2. The proportion was highest in May 2019 (46/2624, 1.75%) and December 2018 (36/2041, 1.76%), which are the final exam periods in the United States.

## Discussion

### Principal Findings

This study was conducted to analyze tweets about the nonmedical use and side effects of methylphenidate. Because there were more than 30,000 tweets mentioning methylphenidate written in a year, it was difficult to classify them manually; therefore, we used the SVM machine learning approach. Similar stimulants, such as Adderall, mixed amphetamine salts, have been studied before [25,29]. An early study using Twitter measured the co-occurrence of nonmedical or side effect terms among 213,633 tweets mentioning Adderall [25]. However, not all Adderall tweets referenced first-hand experience; as the author of that study mentioned, the analysis included 5169 song lyrics [25]. Another recent study automatically detected tweets related to the nonmedical use of Adderall using an SVM approach [29]. However, the SVM classifier had a poor performance: F_1_ score of 0.46, precision of 0.41, and recall of 0.51 [29]. Our study is the first to analyze tweets about the nonmedical use and side effects of methylphenidate and has two main advantages: training first-hand experience and better performance.

In the annotation process, nonmedical use of methylphenidate was identified in 5% of the training dataset, lower than in two previous studies about Adderall (12.9% and 22.6%) [25,29]. This may be due to the popularity of Adderall. In a survey of 4580 college students, three-quarters of those who had engaged in nonmedical use of stimulants over the past year had used Adderall, and one-quarter had taken methylphenidate [7]. In another survey, 54.2% of respondents who abused stimulants used Adderall versus 15% who used methylphenidate [30]. Alternatively, this may be due to stringent standards used in our study: we included only first-hand experience and evaluated whether methylphenidate was administered for medical purposes based on past tweets from the users.

Nonmedical use and side effect terms improved the SVM classifiers the most. The inclusion of personal nouns in the model also significantly improved the classifier. Sentiment scores also contributed to the improvement of the classifier, although they did not capture users’ exact sentiments toward methylphenidate. The previous Adderall study included sentiment analysis in the SVM classifier, which slightly improved the F_1_ scores [29].

The F_1_ scores of SVM classifiers for nonmedical use and side effects were 0.547 and 0.733, respectively. SVM classifiers had low F_1_ scores due to low recall, which may have induced underestimation of nonmedical use and side effects in the test dataset (1.3% and 1.9%, respectively). In particular, the recall of nonmedical use was low because the classifier was built solely on the content of tweets, although the label was annotated by reviewing the users’ previous tweets to see if they had been diagnosed with ADHD. However, because precision was high, tweets classified as *nonmedical use* can be thought of as TP.

In May 2019 and December 2018, the US exams periods, the proportion of tweets related to nonmedical use of methylphenidate was highest. Tweet timing may differ from the time of administration because users sometimes write about past experiences. Nevertheless, higher ratios relative to the other periods may indicate frequent administration of methylphenidate during the exam period for the purpose of improving concentration. The increase in the number of tweets about stimulants in May 2019 and December 2018 was also reported in the Adderall studies [25,29].

### Limitations

The final SVM classifiers had low recall, especially for tweets about nonmedical use. This is because some tweets were ambiguous and previous tweets were not considered in the SVM classifier. If user information or previous tweet information could be included as a feature, it might help to solve the problem of low recall. Another problem is scarcity of positive samples relative to negative samples, despite the use of several methods to resolve the problem of imbalance. The lack of positive data made it impossible to learn about individual side effects, such as sleep disorders and heart problems. Furthermore, the SVM classifier for side effects was not sufficient to detect new side effects because the terms corresponding to known representative side effects were used as features in the supervised learning process.

The frequency of tweets reported in this study does not imply the actual prevalence of nonmedical use or side effects of methylphenidate. First, the study only targeted users of Twitter, which restricts use by individuals under the age of 13 years. Second, the calculated percentage is the percentage of tweets, not the percentage of respondents, as in a general survey. Third, not all Twitter users who take methylphenidate will necessarily write tweets about the drug and among those who do, some may include the drug name and information about nonmedical use or side effects in separate tweets within the same thread. Finally, the study did not take into account various typos in the search terms or non-English tweets.

### Conclusions

This study built SVM classifiers that helped to automatically identify the nonmedical use and side effects of methylphenidate from Twitter. The SVM classifiers had high precision and accuracy but low recall. Information available on Twitter is not available during the prescription process and cannot be identified through electronic medical records. Similar information can be obtained through surveys, as in previous studies, but research using Twitter has the advantage of saving time and cost required for a survey. Future studies should seek to apply this method to other social media platforms.

## Abbreviations

ADHD: attention deficit hyperactivity disorder
FN: false negatives
FP: false positives
SNS: social networking service
SVM: support vector machine
TN: true negatives
TP: true positives
